# Joint effects of folate intake and one-carbon-metabolizing genetic polymorphisms on breast cancer risk: a case-control study in China

**DOI:** 10.1038/srep29555

**Published:** 2016-07-12

**Authors:** Wei-Ping Luo, Bin Li, Fang-Yu Lin, Bo Yan, Yu-Feng Du, Xiong-Fei Mo, Lian Wang, Cai-Xia Zhang

**Affiliations:** 1 Department of Medical Statistics and Epidemiology, School of Public Health, Sun Yat-sen University, Guangzhou 510080, China; 2 Epidemiology Research Unit, the First Affiliated Hospital of Sun Yat-sen University, Guangzhou 510080, China; 3 Nursing Department, the First Affiliated Hospital of Sun Yat-sen University, Guangzhou 510080, China; 4 Department of Vascular Surgery, the First Affiliated Hospital of Sun Yat-sen University, Guangzhou 510080, China

## Abstract

This study aimed to examine the joint effects of folate intake, polymorphisms of 5,10- methylenetetrahydrofolate reductase (*MTHFR),* methionine synthesis reductase (*MTRR*) and methionine synthase (*MTR*) genes and breast cancer risk. A case-control study of 570 consecutively recruited breast cancer cases and 576 controls was conducted in Guangzhou, China. Multifactor dimensionality reduction and logistic regression approach were used to evaluate gene-gene interaction. The covariates were chosen based on comparison of baseline characteristics of cases and controls. Folate intake was found to be inversely associated with breast cancer risk. The *MTRR*rs162036 GG genotype was associated with a decreased risk of breast cancer [adjusted odds ratio (OR) 0.41, 95% confidence interval (CI) 0.20–0.85]. Compared with the wild-type group (*MTRR*rs162036 AA with *MTR*rs1805087 AA) *MTRR*rs162036 AA with *MTR*rs1805087 GA + GG was associated with a decreased risk (OR 0.70, 95% CI 0.48–1.03). With the combined *MTHFR*rs1801131 TT and *MTHFR*rs1801133 GG genotypes as a reference, *MTHFR*rs1801131 TT with *MTHFR*rs1801133 GA + AA was associated with a decreased risk (OR 0.78, 95% CI 0.57 – 1.08) and *MTHFR*rs1801131 GT + GG with *MTHFR*rs1801133 GA + AA was associated with an increased risk (OR 1.35, 95% CI 0.88–2.05). The joint impact of *MTRR*rs162036 and *MTR*rs1805087, *MTHFR*rs1801131 and *MTHFR*rs1801133, folate and *MTHFR*rs1801133 may contribute to breast cancer risk.

Folate in the one-carbon metabolism pathway can influence DNA synthesis, repair, and methylation, which played a critical role in carcinogenesis^1^. Polymorphisms in critical enzymes involved in one-carbon metabolism could also influence the risk of cancer in conjunction with folate consumption^2^. The 5,10-methylenetetrahydrofolate reductase (*MTHFR*) is a key enzyme in folate metabolism, irreversibly catalyzing the conversion of 5,10-methylene tetrahydrofolate (5,10-methylene THF) to 5-methyltetrahydrofolate (5-methyl THF), the primary circulatory form of folate and a carbon donor for remethylation of homocysteine to methionine. The latter is the precursor for *S*-adenosylmethionine, which is the universal methyl donor^3^. Other enzymes, methionine synthase (*MTR*) and methionine synthesis reductase (*MTRR*), are critical enzymes responsible for the biosynthesis of methionine, the precursor for methylation reactions, and the regeneration of THF for nucleotide biosynthesis^4^.

Given the relevance of these mechanisms to carcinogenesis, dietary folate intake and genetic polymorphisms of related genes in the one-carbon metabolism pathway may be associated with the development of breast cancer^5^. However, the epidemiological studies have yielded inconsistent results. Some studies have suggested an inverse association between folate intake and breast cancer risk^6^,^7^,^8^. A recent meta-analysis showed no clear overall association between folate intake or folate level in blood and breast cancer risk^9^. A number of studies have examined *MTHFR* and *MTR* polymorphisms and found that variant genotypes of *MTHFR*rs1801133 GA + AA^10^, *MTHFR*rs1801131GG^11^ and *MTR*rs1805087 GA^2^ were associated with a decreased risk of breast cancer. In addition, several studies have suggested an interaction between *MTHFR*rs1801131 or *MTHFR*rs1801133 and folate intake in breast cancer risk^8^,^11^. It has to be noted that most of the studies focused on the association between single candidate gene polymorphism and breast cancer. However, there were a number of genes involved in one-carbon folate metabolism pathway so that it was limited to study about the single gene polymorphism for evaluating cancer risk susceptibility. Thus, it has been suggested that risk for breast cancer is much better assessed when gene-gene interactions are performed.

Our previous publication^12^ has shown that dietary folate intake was inversely associated with breast cancer risk. The aim of the present case-control study was to explore the associations of *MTHFR*rs1801131, *MTHFR*rs1801133, *MTRR*rs162036, *MTRR*rs1801394 and *MTR*rs1805087 genotype with breast cancer risk, and further investigate the joint effects of folate intake and one-carbon-metabolizing genetic polymorphisms on breast cancer risk among Chinese women.

## Results

The sociodemographic and established breast cancer risk factors of cases and controls are shown in Table 1. Compared to controls, breast cancer cases were more likely to have lower SES, and to regularly consume alcohol. Breast cancer cases were also more likely to have a history of breast cancer in a first-degree relative, to have previous benign breast disease, and to have exposure to passive smoking in their household. The median intake of folate was 193.1 and 214.7 μg/d among cases and controls. Compared with the controls, cases had significantly lower folate intake.

Data for the associations between dietary folate intake, genotypes of related enzymes and breast cancer risk are presented in Table 2. Compared with the lowest quartile of folate intake, the highest intake quartile was inversely associated with the risk of breast cancer (OR 0.39, 95% CI 0.27–0.56) (*P*_trend_ < 0.01). No deviation from the Hardy-Weinberg equilibrium was observed in the controls. *MTHFR*rs1801131 had low linkage disequilibrium with *MTHFR*rs1801133 (*r*^*2*^ = 0.261) and *MTRR*rs1801394 had low linkage disequilibrium with *MTRR*rs162036 (*r*^*2*^ = 0.104). The frequencies of *MTRR*rs162036 AA, GA and GG genotypes were 63.2, 34.9 and 1.9% in cases and 64.4, 30.2 and 5.4% in controls, respectively. Genotypes of *MTRR*rs162036 were not equally distributed in breast cancer cases and controls (*P* = 0.003). With the *MTRR*rs162036 AA genotype as a reference, the GG genotype was associated with a decreased risk of breast cancer (OR 0.41, 95% CI 0.20–0.85). There were no significant associations for other four SNPs.

**Figure 1:**
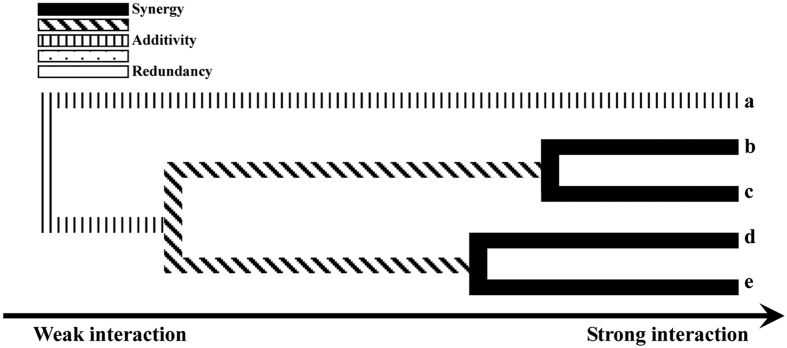
Dendrogram of gene-gene interaction for breast cancer risk. (**a**) *MTRR*rs1801394; (**b**) *MTRR*rs1602036; (**c**) *MTR*rs1805087; (**d**) *MTHFR*rs1801131; (**e**) *MTHFR*rs1801133.

Table 3 summarized the cross-validation consistency and test balance accuracy obtained from MDR analysis of the breast cancer case-control data set, for each number of SNPs evaluated. One four-locus model had a maximum test balance accuracy of 0.5371 and a maximum cross-validation consistency of 10/10 that was significant at the 0.01 level, as determined empirically by permutation testing. Thus, under the null hypothesis of no association, it is highly unlikely that a cross-validation consistency of 10/10 will be observed for this four-locus model. The four-locus model included the polymorphisms of *MTRR*rs162036, *MTR*rs1805087, *MTHFR*rs1801133 and *MTHFR*rs1801131. The interaction dendrogram showed that there were synergistic interactions between *MTHFR*rs1801131 and *MTHFR*rs1801133, and between *MTRR*rs162036 and *MTR*rs1805087 *MTRR*rs1801394 with a vertical line suggested an additive effect (Fig. 1).

**Figure 2:**
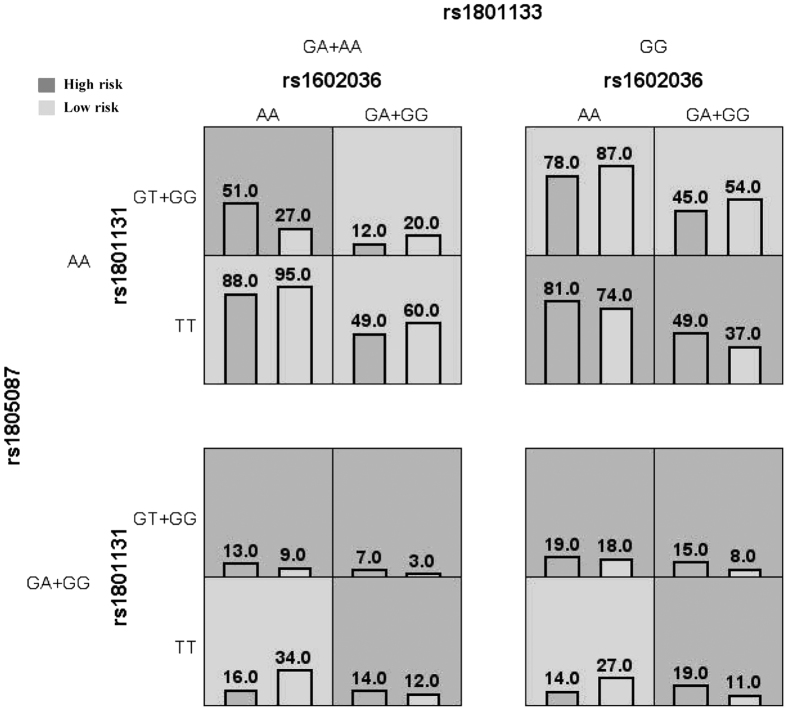
Summary of four-locus genotype combinations associated with high risk and with low risk for breast cancer. Dark gray and light gray boxes presented the high- and low-risk factor combinations, respectively. Left bars within each box represented the cases while the right bars represented the controls. The heights of the bars are proportional to the sum of samples in each group.

The associations between four-locus genotype combinations and the risk of breast cancer are shown in (Fig. 2). There were no obvious trends in the distribution of high risk and low risk groupings among these 16 genotype combinations.

As shown in Table 4, logistic regression analysis showed a significant interaction between *MTRR*rs162036 and *MTR*rs1805087 in breast cancer (*P*_interaction_ = 0.02). Likewise, there was an interaction between *MTHFR*rs1801131 and *MTHFR*rs1801133 in breast cancer risk (*P*_interaction_ = 0.02). However, neither of the Bonferroni correction was statistically significant. When compared with the reference group (*MTRR*rs162036 AA with *MTR*rs1805087 AA genotypes), *MTRR*rs162036 AA with *MTR*rs1805087 GA + GG was associated with a decreased risk of breast cancer (OR 0.70, 95% CI 0.48–1.03) and *MTRR*rs162036 GA + GG with *MTR*rs1805087 GA + GG was associated with an increased risk of breast cancer (OR 1.54, 95% CI 0.95–2.48). With the combined *MTHFR*rs1801131 TT and *MTHFR*rs1801133 GG genotypes as a reference, *MTHFR*rs1801131 TT with *MTHFR*rs1801133 GA + AA was associated with a decreased the risk of breast cancer (OR 0.78, 95% CI 0.57–1.08). Besides, *MTHFR*rs1801131 GT + GG with *MTHFR*rs1801133 GA + AA was associated with an increased risk of breast cancer (OR 1.35, 95% CI 0.88–2.05). All of these ORs were borderline statistically significant.

The joint effects of *MTHFR, MTRR* and *MTR* polymorphisms and dietary folate intake on breast cancer risk are shown in Table 5. The significant interaction was detected for folate intake with *MTHFR*rs1801133 polymorphism, even after the Bonferroni correction. (*P*_interaction_ = 0.01 and Bonferroni *P*_interaction_ = 0.05). Compared with women with *MTHFR*rs1801133 GG and folate intake less than 214.7 μg/d, those having the *MTHFR*rs1801133 GA + AA genotype and folate intake more than 214.7 μg/d was associated with a decreased risk of breast cancer (OR 0.42, 95% CI 0.29–0.61). However, women having *MTHFR*rs1801133 GA + AA genotype and folate intake less than 214.7 μg/d was associated with an increased risk of breast cancer (OR 1.28, 95% CI 0.93–1.77). No significant interaction was found between other genetic polymorphisms and folate consumption in breast cancer risk.

Because household income and educational level were not well-balanced between cases and controls in the present study, a stratification analysis was conducted by these two socioeconomic factors. The results showed that no interaction was observed between socioeconomic status and folate intake and five SNPs (data not shown).

## Discussion

In this study, we confirmed that dietary folate intake was inversely associated with breast cancer risk. We observed an inverse association between the *MTRR*rs162036 GG genotype and breast cancer risk. Both MDR approach and logistic regression analysis supported that there were gene-gene interactions between *MTRR*rs162036 and *MTR*rs1805087 and between *MTHFR*rs1801131 and *MTHFR*rs1801133 in breast cancer risk. Interaction was also observed between consumption of folate and *MTHFR*rs1801133.

It has been suggested that genetic polymorphisms in folate metabolic enzyme genes could influence breast cancer risk^13^. Some studies found that there was no association between *MTHFR*rs1801131^8^,^14^,^15^, *MTHFR*rs1801133^7^,^11^,^14^,^15^,^16^, *MTRR*rs1801394^2^,^14^,^16^ and *MTR*rs1805087^15^ and breast cancer risk. In the present study, we did not find an overall reduced risk of breast cancer associated with *MTHFR*rs1801131, *MTHFR*rs1801133, *MTRR*rs1801394 and *MTR*rs1805087 genotypes, which was consistent with previous studies. Some studies showed that *MTRR*rs162036 GG polymorphism was not associated with the incidence of colorectal cancer^17^, cervical cancer^18^ or gastric cancer^19^ when using the *MTRR*rs162036 AA genotype as a reference. However, the present study, the first study assessing the relationship between *MTRR*rs162036 polymorphism and breast cancer risk, showed that the homozygous GG genotype was a protective factor for breast cancer. The possible mechanism might be that the function of *MTRR* is essential for providing methyl groups. It is highly likely that enzymatic variants due to functional polymorphisms may alter DNA methylation, which would greatly affect carcinogenesis^20^.

As more and more studies evaluate risk associated with multiple genes factors, it has become clear that traditional logistic regression analysis could not deal with the dimensionality problem very effectively and is not adequate for modeling complex multi-factor interactions^21^. For this reason, we utilized the recently developed MDR approach to assess and interpret potential interactions. This approach improves statistical power to efficiently identify potential gene-gene interactions. However, the MDR approach does not allow for adjustment of confounding factors but logistic regression analysis does. Therefore, the gene-gene interactions were identified by both the logistic regression analysis and the MDR approach in our study. The results of the MDR analysis showed that *MTRR*rs162036, *MTHFR*rs1801131, *MTHFR*rs1801133 and *MTR*rs1805087 was the best four factor model with the maximum test balance accuracy and cross-validation consistency compared with other models. However, the other models were not likely to generalize to independent datasets due to the lower test balance accuracy and cross-validation consistency. Meanwhile, the interaction dendrogram showed that there were interactions between *MTHFR*rs1801131 and *MTHFR*rs1801133 and between *MTRR*rs162036 and *MTR*rs1805087. The logistic regression analysis further validated the results of MDR analysis. To the best of our knowledge, no previous studies have investigated the interactions between genetic polymorphisms in breast cancer risk using both MDR analysis and logistic regression analysis. However, in terms of bladder cancer, a case-control study conducted in America showed that applying these two methods might facilitate the identification of gene-gene interactions^22^.

Some studies have analyzed the interaction between *MTHFR*rs1801131 and *MTHFR*rs1801133 by logistic regression analysis, but have yielded controversial results. Three studies^23^,^24^,^25^ showed that there was no significant interaction between *MTHFR*rs1801131 and *MTHFR*rs1801133 in breast cancer risk. On the other hand, two studies^10^,^26^ found that the presence of *MTHFR*rs1801131 GG and/or *MTHFR*rs1801133 AA was associated with a decreased risk of breast cancer compared with compound wild type subjects, whereas three studies^11^,^27^,^28^ found an increased risk. Our results confirmed the detrimental interaction between the *MTHFR*rs1801131 GT + GG and *MTHFR*rs1801133 GA + AA in breast cancer risk. The mechanism might be that the combined effect of *MTHFR*rs1801131 GT + GG and *MTHFR*rs1801133 GA + AA have lowered *MTHFR* enzyme activity, elevated homocysteine, decreased plasma folate levels and increased the risk of cancer^29^.

No previous studies have examined the interaction between *MTRR*rs162036 and *MTR*rs1805087 in any cancer. Our study found that the combination of *MTRR*rs162036 GA + GG and *MTR*rs1805087 GA + GG was associated with an increased risk of breast cancer compared with *MTRR*rs162036 AA with *MTR*rs1805087 AA. Although the Bonferroni-corrected *P*_interaction_ were above 0.05, Bonferroni method is often considered to be overly conservative^30^. The possible mechanism for the interaction might be that the polymorphism of *MTR*rs1805087 affected the enzymatic activity of *MTR* and may affect DNA methylation. It was reported that *MTR*rs1805087 GA + GG was associated with both global genomic hypomethylation and low level of hypermethylated CpG islands within promoters of tumor suppressor genes in individuals with kinds of cancers^31^. Moreover, *MTRR*rs162036 GA + GG might lead to the decreased activity of *MTR* because *MTRR* serves to maintain *MTR* in its active state^32^.

It is biologically plausible that folate-related gene–nutrient interactions might play a role in breast cancer risk. The studies in Brazil^13^, America^33^ and China^8^ found that there was a significant interaction between folate intake and *MTHFR*rs1801133 polymorphism in breast cancer risk (*P* < 0.05). Our study also found an interaction between folate consumption and *MTHFR*rs1801133 polymorphism in breast cancer risk. *MTHFR*rs1801133 GA + AA genotype was associated with an increased risk of breast cancer among women with folate intake less than 214.7 μg/d and a decreased risk of breast cancer among women with folate intake more than 214.7 μg/d. It is well known that *MTHFR* is a key enzyme in folate metabolism, irreversibly catalyzing the conversion of 5,10-methylene THF to 5-methyl THF, the primary circulatory form of folate. The 5,10-methylene THF reduces the incorrect inclusion of uracil in the DNA, leading to double-strand breakages during excision of uracil and increased chromosome instability, which is a phenomenon usually associated to carcinogenesis^34^. Moreover, the lower activity of variant genotypes may increase the risk of breast cancer at low levels of dietary folate since less 5-methyl THF is made available for DNA methylation^13^. Whereas, the compensation of sufficient folate intake could reverse DNA hypomethylation^35^. However, no significant interactions were found between folate intake and any polymorphisms of *MTHFR*rs1801131, *MTRR*rs162036, *MTRR*rs1801394 and *MTR*rs1805087 in the risk of breast cancer.

The present study had some methodological strength. This is the first study simultaneously investigating the role of two *MTRR* polymorphisms in modulating the susceptibility to breast cancer. Moreover, this is the first epidemiological study that investigated the interaction between genetic polymorphisms of related enzymes in breast cancer risk by MDR approach. Furthermore, a validated FFQ was used to assess the frequency and portion size of dietary folate intake. We had a wide range of potential confounders including dietary and non-dietary factors and were able to adjust for them in the analyses.

Our study had some limitations. First, the results obtained in this study could be affected by sources of selection bias that commonly emerged in case-control study. However, the genotype frequencies of the SNPs among our controls were consistent with those derived from the Hardy-Weinberg equilibrium. Moreover, the high participation rate (93 and 94% for cases and controls, respectively) also helped to minimize this bias. Second, recall bias is also of concern in case-control studies. Controls did not have a malignant problem and the recall of their past food intake might be different from the cases. To reduce this bias, we tried to interview the cases as soon as the diagnosis was made and food photographs were provided as visual aids. Third, misclassification in genotyping status is unavoidable. However, cases and controls were analyzed in the same plate by laboratory personnel who were blinded to case-control status and there was no discrepancy between repeated analyses. Moreover, the genotyping call rate for each SNP was 100%. Therefore, any influence of genotyping misclassification on the present findings was likely to be minimal. Fourth, it was difficult to interpret the MDR model. A consistent trend of high risk or low risk cells across a series of rows or columns may indicate that a particular locus has a main effect^36^. However, in the present study, MDR analysis showed that there were no obvious trends in the distribution of high risk and low risk groupings across the four-locus model. Fifth, socioeconomic factors (income and educational level) were not well-balanced between the cases and controls in the present study. However, stratification analysis by socioeconomic factors observed no interaction between socioeconomic factors and folate intake and five SNPs. This suggested that the unbalanced socioeconomic factors would not modify the results for the total study subjects. Sixth, the sample size was small in the subset analysis. Due to the high number of models evaluated and tests performed, the false-positive results might occur. Therefore, the current results should be interpreted with caution and further studies with larger sample size are needed to confirm these associations. Seventh, the present study did not assess folate supplemental intake. However, most Chinese women consume natural (unfortified and unprocessed) foods, and they seldom take vitamin supplements including folate supplementation. Serum folate level was also not measured in the present study. Therefore, further studies with blood folate level measurement will help strengthen the understanding the association between folate and breast cancer risk.

In conclusion, our study showed that the *MTRR*rs162036 GG genotype was associated with a decreased risk of breast cancer. Significant gene-gene interactions were found between *MTRR*rs162036 and *MTR*rs1805087, *MTHFR*rs1801131 and *MTHFR*rs1801133. And there was an interaction between folate intake and *MTHFR* rs1801133 gene variants in relation to risk for breast cancer. Breast cancer is a multi-factorial complex disease. More emphasis should be placed on the detection and characterization of multiple genetic and environmental interactions of breast cancer to predict high-risk individuals for the personalized prevention and treatment of breast cancer.

## Material and Methods

### Study subjects

This is an ongoing case-control study beginning in September 2011. Potential cases were recruited among patients who admitted to the surgical units of three teaching and general hospitals in Guangzhou from September 2011 to September 2014. Inclusion criteria were female subjects aged 25–70 years and natives of the province of Guangdong or having lived in Guangdong for at least 5 years, with incident, primary, histological confirmed breast cancer diagnosed no more than 3 months before the interview. Women were excluded if they could not understand or speak Mandarin/Cantonese or with prior history of breast cancer or other cancers. A total of 613 eligible cases were identified and 570 were interviewed, yielding a participation rate of 93%.

Controls were patients with no history of cancer and admitted to the same hospitals during the same time period as the cases. They were frequency matched by age (5-year interval) to the case patients. They were selected from the departments of Ophthalmology, Plastic and Reconstructive Surgery, Vascular Surgery, Ear-Nose-Throat, and Orthopedics and Microsurgery. In total, 576 controls out of 613 eligible controls were successfully interviewed (94% participation rate).

The present study was approved by the ethical committee of School of Public Health of Sun Yat-sen University. Written informed consent was obtained from all participants prior to the interview and the methods were carried out in accordance with the approved guidelines.

### Data collection

Trained interviewers conducted face-to-face interviews using a structured questionnaire to collect information on sociodemographic factors, body weight and height, lifestyle factors (*e.g.* active and passive smoking, alcohol drinking, and physical activity), menopausal status, reproductive history and family history of cancer. In this study, regular smokers were defined as someone smoking at least one cigarette a day for more than 6 consecutive months. Passive smoking meant to be exposed to others’ tobacco smoke for at least 5 min/day in the previous 5 years. Regular drinking was defined as alcohol drinking at least once per week in the past year. Postmenopausal status was defined as at least 12 months since the last menstrual cycle. Body mass index (BMI) was calculated by dividing weight (kg) by height squared (m^2^). The socioeconomic status (SES) was obtained by accumulating the total scores of education, income and occupation and divided into lower, middle and upper groups according to the manual of socioeconomic status scale^37^. Relevant medical information, medical diagnosis and histological findings were abstracted from the hospital medical records.

### Dietary assessment

Dietary information was obtained from an 81-item interviewer-administered food frequency questionnaire (FFQ) covering the habitual diet of participants during the previous year. Food photographs were used to help participants quantify the portions consumed. Information on frequency of intake and portion size was used to calculate the amount of each food item consumed on average (g/d). Daily nutrients intakes were estimated using the China Food Composition Table^38^. Dietary folate intake was calculated by summing the product of the frequency of consumption, usual portion consumed and folate content of each food item.

### Genotype of polymorphisms

Blood samples were collected immediately after admission to the hospital for cases or after the interview for controls and were stored at −80 °C until analysis. Genomic DNA samples were extracted from the peripheral blood using a TIANamp Genomic DNA Kit (TIANGEN BIOTECH, Beijing, China) according to the manufacturer’s instructions. We selected five single nucleotide polymorphisms (SNPs) in three genes (*MTHFR, MTRR* and *MTR*). We chose SNPs with an expected minor allele frequency >5% that showed previous evidence of associations with cancer risk or had possible functional significance. Genotyping of 5 polymorphisms in *MTHFR*rs1801131, *MTHFR*rs1801133, *MTRR*rs162036, *MTRR*rs1801394 and *MTR*rs1805087 was performed using a custom-by-design 48-Plex SNPscan Kit (Genesky Biotechnologies Inc., Shanghai, China) as previously described^39^. This kit was developed according to patented SNP genotyping technology by Genesky Biotechnologies Inc., which was based on double ligation and multiplex fluorescence PCR. For quality control, cases and controls were analyzed in the same plate by laboratory personnel who were blinded to case and control status. We have genotyped 570 cases and 576 controls and all of them were included in the study. The genotyping call rate for each SNP was 100%. Fifty-five repeated samples were taken from the cases and controls and reached the duplication rate of 4.58%. There was no discrepancy between repeated analyses.

### Statistical analysis

Dietary folate intake was adjusted for total energy intake using the residual method^40^ and then categorized into quartiles based on the distribution among the controls. The lowest quartile with the lower folate intake served as the reference group in the analyses. Odds ratios (ORs) and 95% confidence intervals (95% CIs) summarizing the association between breast cancer risk and folate intake were calculated by using unconditional logistic regression after adjustment for SES, marital status, family history of cancer, history of benign breast disease, live births, months of breast-feeding, passive smoking from husband, alcohol drinking, physical activity, menopausal status, BMI, nulliparous and oral contraceptive use. To calculate the *P* values for trend of folate intake, folate intake was regarded as a continuous variable after coding as 1 (below Q1), 2 (Q2-Q3), 3 (Q3-Q4) and 4 (above Q4). When the intake was categorized into two groups, it was coded as 1 (below median) and 2 (above median). To calculate the *P* values for the trend of each SNP, the genotypes was regarded as a continuous variable after coding as 1 (wild-type), 2 (hybrid-type) and 3 (mutant-type)^41^. All of the aforementioned statistical analyses were performed using SPSS 13.0 (SPSS Inc., Chicago, IL, USA).

Hardy-Weinberg equilibrium (HWE) was used to assess whether genotypes fell within a standard distribution. Deviation from the HWE in genotype frequencies was assessed with chi-square test. To test for deviation from HWE, comparisons were made between observed heterozygosity (*H*_*o*_) and expected heterozygosity (*H*_*e*_) using exact tests implemented by Microsoft Excel. To increase statistical power, respective genotypes were combined into two groups when the polymorphisms were less frequent. GT and GG genotype groups of *MTHFR*rs1801131, GA and AA genotype groups of *MTHFR*rs1801133, GA and GG genotype groups of *MTRR*rs162036, GA and GG genotype groups of *MTRR*rs1801394 and GA and GG genotype groups of *MTR*rs1805087 were combined, respectively. Homozygous genotypes of *MTHFR*rs1801131 TT, *MTHFR*rs1801133 GG, *MTRR*rs162036 AA, *MTRR*rs1801394 AA and *MTR*rs1805087 AA were considered as the reference group. Linkage disequilibrium between the two *MTHFR* polymorphisms and the two *MTRR* polymorphisms were obtained with Haploview 4.2 (Cambridge, MA, USA) and tight linkage disequilibrium was defined by *r*^*2*^ > 0.8^42^.

Multi-factor Dimensionality Reduction (MDR) analysis was performed to evaluate the interactions between genetic polymorphisms and create the interaction dendrogram and graph by MDR 3.0.2 software. The software gives number of output parameters like cross-validation consistency, test balance accuracy for different interactions and single best model is identified as interaction that had maximum consistency and accuracy. In the interaction dendrogram, a black or diagonal line connecting two polymorphisms suggests a synergistic or nonadditive relationship while a vertical line indicates independence or additivity. A spotted or white line suggests a loss of information which can be interpreted as redundancy or correlation (*e.g.* linkage disequilibrium). Statistical significance of the model was evaluated using a 1000-fold permutation test. Meanwhile, logistic regression approach was used to evaluate the interaction between *MTHFR, MTRR* or *MTR* genotype in breast cancer risk. The combined effects of *MTHFR, MTRR* or *MTR* polymorphisms were assessed by adding the multiplicative interaction product (genotype × genotype) into the final model as an indicator variable. Similarly, the multiplicative interaction product [dietary folate intake values below and above the median (<214.7 *versus* ≥ 214.7 μg/d) × genotype] was added into the final model to identify the joint effects of dietary folate intake and each SNPs. Stratified analyses by socioeconomic factors (income and educational level) were also conducted. In this study, all *P* values are two sided and statistical significance was determined at the *P* value less than 0.05 level. The Bonferroni method was used to adjust the *P* value for multiple comparisons.

## Additional Information

**How to cite this article**: Luo, W.-P. *et al*. Joint effects of folate intake and one-carbon-metabolizing genetic polymorphisms on breast cancer risk: a case-control study in China. *Sci. Rep.* **6**, 29555; doi: 10.1038/srep29555 (2016).

## Figures and Tables

**Table 1:** Sociodemographic characteristics and selected risk factors of breast cancer in the study population.

Variables	Cases (n = 570)	Controls (n = 576)	*P*^a^
Age (mean ± SD, years)	47.54 ± 9.29	48.0 ± 9.44	0.36
Age (n, %)			0.73
25–30	14 (2.5)	17 (3.0)	
31–35	45 (7.9)	44 (7.6)	
36–40	89 (15.6)	72 (12.5)	
41–45	113 (19.8)	118 (20.5)	
46–50	128 (22.5)	122 (21.2)	
51–55	60 (10.5)	63 (10.9)	
56–60	68 (11.9)	85 (14.8)	
61–65	41 (7.2)	38 (6.6)	
66–70	12 (2.1)	17 (3.0)	
Marital status (n, %)			0.21
Married	537 (94.2)	532 (92.4)	
Unmarried/divorced/widowed	33 (5.8)	44 (7.6)	
Socioeconomic status, SES (n, %)			<0.01
Lower	168 (29.5)	138 (24.0)	
Middle	262 (46.0)	247 (42.9)	
Upper	140 (24.6)	191 (33.2)	
Physical activity (exercise for health; n, %)			0.18
Never	354 (62.1)	327 (56.8)	
Seldom	109 (19.1)	122 (21.2)	
Often	107 (18.8)	127 (22.0)	
Menopausal status (n, %)			0.12
Premenopausal	380 (66.7)	371 (64.4)	
Postmenopausal	190 (33.3)	205 (35.6)	
Regular smoker (n, %)	6 (1.1)	5 (0.9)	0.75
Passive smoking from husband (n, %)	284 (49.8)	224 (38.9)	<0.01
Regular drinker (n, %)	48 (8.4)	29 (5.0)	0.02
Body mass index, BMI (mean ± SD)	23.05 ± 3.28	22.69 ± 3.19	0.06
Age at menarche (mean ± SD, years)	14.60 ± 1.91	14.49 ± 1.76	0.32
Nulliparous (n, %)	26 (4.6)	35 (6.1)	0.25
Age at first live birth (n, %)^b^	25.60 ± 3.59	25.58 ± 3.26	0.91
Breastfeeding (n, %)	471 (82.6)	479 (83.2)	0.81
Months of breastfeeding (n, %)^b^			0.06
Never	73 (13.4)	62 (11.5)	
1–3	29 (5.3)	30 (5.5)	
4–11	125 (23.0)	163 (30.1)	
≥ 12	317 (58.3)	286 (52.9)	
Benign breast disease (n, %)	200 (35.1)	134 (23.3)	<0.01
Ever used an oral contraceptive (n, %)	40 (7.0)	28 (4.9)	0.12
First-degree relative with cancer (n, %)	81 (14.2)	58 (10.1)	0.03
Total energy intake (mean ± SD, kcal/d)	1404.57 ± 381.14	1427.63 ± 363.78	0.30
Intake of folate (median, 25^th^–75^th^ percentile, μg/d)^c^	193.1, 168.2–225.4	214.7, 185.1–247.0	<0.01

^a^Continuous variables were evaluated using *t*-tests or Wilcoxon rank-sum tests. Categorical variables were evaluated using χ^2^-tests.

^b^Among parous women.

^c^Dietary folate intake was adjusted for total energy intake using the residual method.

**Table 2:** Association between genotypes of related enzymes and dietary folate intake and breast cancer risk.

	Cases (n, %)	Controls (n, %)	Crude OR (95% CI)	Adjusted OR (95% CI)^a^
Folate intake (μg/d)				
Q1 (<185.1)	227 (39.8)	144 (25.0)	1.00 (reference)	1.00 (reference)
Q2 (185.1–214.7)	166 (29.2)	144 (25.0)	0.73 (0.54−0.99)	0.77 (0.56−1.06)
Q3 (214.7–247.0)	97 (17.0)	144 (25.0)	0.43 (0.31−0.60)	0.46(0.33−0.66)
Q4 (>247.0)	80 (14.0)	144 (25.0)	0.35 (0.25–0.50)	0.39 (0.27–0.56)
*P*_trend_			<0.01	<0.01
*MTHFR* rs1801131				
TT (wild-type)	330 (57.9)	350 (60.8)	1.00 (reference)	1.00 (reference)
GT	214 (37.5)	196 (34.0)	1.16 (0.91−1.48)	1.20 (0.93−1.55)
GG	26 (4.6)	30 (5.2)	0.92 (0.53−1.59)	0.98 (0.55−1.73)
*P*_trend_			0.52	0.36
GT + GG	240 (42.1)	226 (39.2)	1.13 (0.89−1.43)	1.17 (0.91−1.50)
*MTHFR* rs1801133				
GG (wild-type)	320 (56.1)	316 (54.9)	1.00 (reference)	1.00 (reference)
GA	215 (37.7)	215 (37.3)	0.99 (0.77−1.26)	0.98 (0.76−1.27)
AA	35 (6.1)	45 (7.8)	0.77 (0.48−1.23)	0.84 (0.51−1.38)
*P*_trend_			0.42	0.58
GA + AA	250 (43.9)	260 (45.1)	0.95 (0.75−1.20)	0.96 (0.75−1.22)
*MTRR* rs162036				
AA (wild-type)	360 (63.2)	371 (64.4)	1.00 (reference)	1.00 (reference)
GA	199 (34.9)	174 (30.2)	1.18 (0.92−1.51)	1.24 (0.95−1.62)
GG	11 (1.9)	31 (5.4)	0.37 (0.18−0.74)	0.41 (0.20−0.85)
*P*_trend_			0.51	0.86
GA + GG	210 (36.8)	205 (35.6)	1.06 (0.83−1.34)	1.12 (0.87−1.44)
*MTRR* rs1801394				
AA (wild-type)	313 (54.9)	314 (54.5)	1.00 (reference)	1.00 (reference)
GA	216 (37.9)	219 (38.0)	0.99 (0.78−1.26)	0.97 (0.75−1.25)
GG	41 (7.2)	43 (7.5)	0.96 (0.61−1.51)	0.91 (0.56−1.47)
*P*_trend_			0.86	0.67
GA + GG	257 (45.1)	262 (45.5)	0.98 (0.78−1.24)	0.96 (0.75−1.22)
*MTR* rs1805087				
AA (wild-type)	453 (79.5)	454 (78.8)	1.00 (reference)	1.00 (reference)
GA	112 (19.6)	112 (9.4)	1.00 (0.75−1.34)	1.00 (0.74−1.36)
GG	5 (0.9)	10 (1.7)	0.50 (0.17−1.48)	0.55 (0.18−1.67)
*P*_trend_			0.57	0.63
GA + GG	117 (20.5)	122 (11.1)	0.96 (0.72−1.28)	0.97 (0.72−1.31)

Q, quartile; CI, confidence interval; OR, odds ratio.

^a^Odds ratio adjusted for age, marital status, SES, family history of cancer, history of benign breast disease, live births, months of breast-feeding, passive smoking from husband, alcohol drinking, physical activity, menopausal status, BMI, nulliparous and oral contraceptive use.

**Table 3:** Summary of multifactor dimensionality reduction (MDR) models for gene-gene interactions in breast cancer.

No. of factors	Best candidate model	Test balance accuracy	Cross-validation consistency	*P*-value
One factor	*MTHFR* rs1801131	0.4822	7/10	0.32
Two factors	*MTRR* rs162036	0.4974	5/10	0.02
	*MTR* rs1805087			
Three factors	*MTHFR* rs1801131	0.4926	5/10	<0.01
	*MTHFR* rs1801133			
	*MTR* rs1805087			
Four factors^a^	*MTRR* rs162036	0.5371	10/10	<0.01
	*MTR* rs1805087			
	*MTHFR* rs1801131			
	*MTHFR* rs1801133			
Five factors	*MTRR* rs162036	0.5301	10/10	<0.01
	*MTR* rs1805087			
	*MTHFR* rs1801131			
	*MTHFR* rs1801133			
	*MTRR* rs1801394			

^a^Best model predicted for breast cancer risk with highest cross-validation consistency and maximum test balance accuracy.

**Table 4:** Associations between gene-gene interactions and breast cancer risk by logistic regression analysis CI, confidence interval; OR, odds ratio.

Genotypes	Genotypes	Cases (n)	Controls (n)	Crude OR (95% CI)	Adjusted OR (95% CI)^a^	*P*_interaction_	Bonferroni *P*_interaction_
*MTHFR* rs1801131	*MTRR* rs162036					0.18	0.90
TT (wild-type)	AA (wild-type)	199	230	1.00 (reference)	1.00 (reference)		
	GA + GG	131	120	1.26 (0.92–1.72)	1.35 (0.97–1.88)		
GT + GG	AA (wild-type)	161	141	1.32 (0.98–1.77)	1.39 (1.02–1.89)		
	GA + GG	79	85	1.07 (0.75–1.54)	1.18 (0.80–1.72)		
*MTHFR* rs1801131	*MTRR* rs1801394					0.91	1.00
TT (wild-type)	AA (wild-type)	184	193	1.00 (reference)	1.00 (reference)		
	GA + GG	146	157	0.98 (0.72–1.32)	0.90 (0.65–1.23)		
GT + GG	AA (wild-type)	129	121	1.12 (0.81–1.54)	1.09 (0.78–1.53)		
	GA + GG	111	105	1.11 (0.79–1.55)	1.14 (0.80–1.61)		
*MTHFR* rs1801131	*MTR* rs1805087					0.30	1.00
TT (wild-type)	AA (wild-type)	267	266	1.00 (reference)	1.00 (reference)		
	GA + GG	63	84	0.75 (0.52–1.08)	0.77 (0.52–1.13)		
GT + GG	AA (wild-type)	186	188	0.99 (0.76–1.28)	1.03 (0.78–1.36)		
	GA + GG	54	38	1.42 (0.90–2.22)	1.45 (0.91–2.32)		
*MTHFR* rs1801133	*MTRR* rs162036					0.11	1.00
GG (wild-type)	AA (wild-type)	192	206	1.00 (reference)	1.00 (reference)		
	GA + GG	128	110	1.25 (0.91–1.72)	1.34 (0.95–1.88)		
GA + AA	AA (wild-type)	168	165	1.09 (0.82–1.46)	1.11 (0.82–1.51)		
	GA + GG	82	95	0.93 (0.65-1.32)	0.99 (0.68-1.44)		
*MTHFR* rs1801133	*MTRR* rs1801394					0.75	1.00
GG (wild-type)	AA (wild-type)	176	171	1.00 (reference)	1.00 (reference)		
	GA + GG	144	145	0.97 (0.71–1.32)	0.96 (0.69–1.34)		
GA + AA	AA (wild-type)	137	143	0.93 (0.68–1.28)	0.96 (0.69–1.35)		
	GA + GG	113	117	0.94 (0.67–1.31)	0.91 (0.65–1.30)		
*MTHFR* rs1801133	*MTR* rs1805087					0.40	1.00
GG (wild-type)	AA (wild-type)	253	252	1.00 (reference)	1.00 (reference)		
	GA + GG	67	64	1.04 (0.71–1.53)	1.07 (0.72–1.61)		
GA + AA	AA (wild-type)	200	202	0.99 (0.76–1.28)	1.00 (0.76–1.32)		
	GA + GG	50	58	0.86 (0.57-1.30)	0.86 (0.55-1.33)		
*MTRR* rs162036	*MTRR* rs1801394					0.72	1.00
AA (wild-type)	AA (wild-type)	164	172	1.00 (reference)	1.00 (reference)		
	GA + GG	196	199	1.03 (0.77–1.38)	1.01 (0.74–1.38)		
GA + GG	AA (wild-type)	149	142	1.10 (0.80–1.51)	1.15 (0.83–1.61)		
	GA + GG	61	63	1.02 (0.67–1.53)	1.06 (0.68–1.63)		
*MTRR* rs1801394	*MTR* rs1805087					0.14	1.00
AA (wild-type)	AA (wild-type)	242	251	1.00 (reference)	1.00 (reference)		
	GA + GG	71	63	1.17 (0.80–1.71)	1.14 (0.77–1.71)		
GA + GG	AA (wild-type)	211	203	1.08 (0.83–1.40)	1.04 (0.79–1.36)		
	GA + GG	46	59	0.81 (0.53–1.24)	0.80 (0.51–1.26)		
*MTRR* rs162036	*MTR* rs1805087					0.02	0.20
AA (wild-type)	AA (wild-type)	298	283	1.00 (reference)	1.00 (reference)		
	GA + GG	62	88	0.67 (0.47–0.96)	0.70 (0.48–1.03)		
GA + GG	AA (wild-type)	155	171	0.86 (0.66–1.13)	0.94 (0.70–1.25)		
	GA + GG	55	34	1.54 (0.97–2.43)	1.54 (0.95–2.48)		
*MTHFR* rs1801131	*MTHFR* rs1801133					0.02	0.20
TT (wild-type)	GG (wild-type)	163	149	1.00 (reference)	1.00 (reference)		
	GA + AA	167	201	0.76 (0.56–1.03)	0.78 (0.57–1.08)		
GT + GG	GG (wild-type)	157	167	0.86 (0.63–1.17)	0.91 (0.65–1.26)		
	GA + AA	83	59	1.29 (0.86–1.92)	1.35 (0.88–2.05)		

^a^Odds ratio adjusted for age, marital status, SES, family history of cancer, history of benign breast disease, live births, months of breast-feeding, passive smoking from husband, alcohol drinking, physical activity, menopausal status, BMI, nulliparous and oral contraceptive use.

**Table 5:** Associations between gene-nutrition interactions and breast cancer risk by logistic regression analysis.

Gene	Genotypes	Folate intake below median (<214.7 μg/d)		Folate intake above median (≥214.7 μg/d)		*P*_trend_^b^	*P*_*interaction*_	Bonferroni *P*_interaction_
		Case/Control	Adjusted OR (95% CI)^a^	Case/Control	Adjusted OR (95% CI)^a^			
*MTHFR* rs1801131							0.46	1.00
	TT (wild-type)	232/171	1.00 (reference)	98/179	0.44 (0.31-0.62)	<0.01		
	GA + AA	161/117	1.07 (0.78–1.48)	79/109	0.58 (0.40–0.84)	<0.01		
	*P*_trend_^c^		0.66		0.18			
*MTHFR* rs1801133							0.01	0.05
	GG (wild-type)	217/174	1.00 (reference)	103/142	0.66 (0.47–0.92)	0.04		
	GA + AA	176/114	1.28 (0.93–1.77)	74/146	0.42 (0.29–0.61)	<0.01		
	*P*_trend_^c^		0.17		0.02			
*MTRR* rs162036							0.29	1.00
	AA (wild-type)	249/179	1.00 (reference)	111/192	0.44 (0.32–0.60)	<0.01		
	GA + GG	144/109	0.98 (0.71–1.36)	66/96	0.57 (0.38–0.84)	0.01		
	*P*_trend_^c^		0.98		0.14			
*MTRR* rs1801394							0.96	1.00
	AA (wild-type)	211/157	1.00 (reference)	102/157	0.48 (0.34–0.68)	<0.01		
	GA + GG	182/131	0.96 (0.70–1.31)	75/131	0.46 (0.32–0.66)	<0.01		
	*P*_trend_^c^		0.76		0.95			
*MTR* rs1805087							0.11	0.55
	AA (wild-type)	305/231	1.00 (reference)	148/223	0.53 (0.40–0.71)	<0.01		
	GA + GG	88/57	1.17 (0.79–1.73)	29/65	0.37 (0.23–0.61)	<0.01		
	*P*_trend_^c^		0.46		0.17			

CI, confidence interval; OR, odds ratio.

^a^Odds ratio adjusted for age, marital status, SES, family history of cancer, history of benign breast disease, live births, months of breast-feeding, passive smoking from husband, alcohol drinking, physical activity, menopausal status, BMI, nulliparous and oral contraceptive use.

^b^Test for linear trend was calculated based on the estimates for folate intake category (coded 1, 2).

^c^Test for linear trend was calculated based on the estimates for SNP genotypes category (coded 1, 2).
